# Simple Thermal-Efficiency Model for CMOS-Microhotplate Design

**DOI:** 10.6028/jres.111.020

**Published:** 2006-06-01

**Authors:** Jon Geist, M. Yaqub Afridi, Ankush Varma, Allen R. Hefner

**Affiliations:** National Institute of Standards and Technology, Gaithersburg, MD 20899

**Keywords:** CMOS, low-voltage, MEMS, microhotplate, model, sub-micron

## Abstract

Simple, semi-empirical, first-order, analytic approximations to the current, voltage, and power as a function of microhotplate temperature are derived. To lowest order, the voltage is independent of, and the power and current are inversely proportional to, the length of the microhotplate heater legs. A first-order design strategy based on this result is described.

## 1. Introduction

A Complementary Metal-Oxide Semiconductor (CMOS) microhotplate-based gas-sensor SoC (System on a Chip) currently under development [2] requires sub-micron integrated-circuit (IC) technology to provide the desired level of performance in a practical-size IC die. A precursor for the microhotplate and the associated signal-processing and control circuits will be fabricated simultaneously in the same standard-CMOS IC-foundry technology. The die will then be further processed (post-processing) to convert the precursor for the microhotplate into a functional microhotplate. [3] This is accomplished by cutting open areas through the CMOS dielectric to expose the silicon substrate, which defines the edges of the microhotplate. Etching the exposed silicon then undercuts and suspends the microhotplate over an etch pit in the silicon substrate. Suspension of the microhotplate over the etch pit provides the thermal isolation necessary to allow the microhotplate to reach temperatures as high as 500 °C with practical power levels and without significant heating of the die on which it is located.

Previous development work [4] on CMOS microhotplate-based gas sensors was carried out by using a mixed-signal technology that allowed on-chip control of heater voltages as large as 13 V. However, in order to obtain sufficient functionality, it will be necessary to produce the SoC in a submicron technology that can provide no more than about 3 V to drive the microhotplate heater. Therefore, efficient use of the available voltage to increase the temperature will become a much more important consideration.

The purpose of this paper is to describe a simple analytic model of the electro-thermal performance of a microhotplate that is suitable for first order design of microhotplates to meet specific temperature, voltage, current, and power requirements. General results are then derived from the model and a first-order design strategy based on these results is presented. This information should not be needed by SoC designers who wish to incorporate a microhotplate into an SoC, but is expected to be useful to intellectual-property (IP) designers who wish to produce microhotplate-based virtual components (VC), which will encapsulate the microhotplate and associated control and signal-processing circuits in a digital interface, for use in SoC designs. It is expected that an empirically improved and extended model will be incorporated into the simulation capability included with the VC.

The remaining sections of this paper 1) outline the derivation of expressions for the heater current, voltage, and power as a function of the microhotplate temperature based on the assumption that the microhotplate properties are independent of temperature, 2) describe an approximation to correct for the dependence of the heater resistance on temperature, 3) describe a microhotplate-design strategy based on this model, 4) illustrate the use of this model to design a microhotplate to reach 500 °C with a heater voltage of 3 V in a typical 0.5 µm technology, 5) compare the predicted and measured performance of a microhotplate fabricated according to the design developed from the model, and 6) summarize general conclusions arising from this work.

## 2. Constant Thermal Properties

**Fig. 1:**
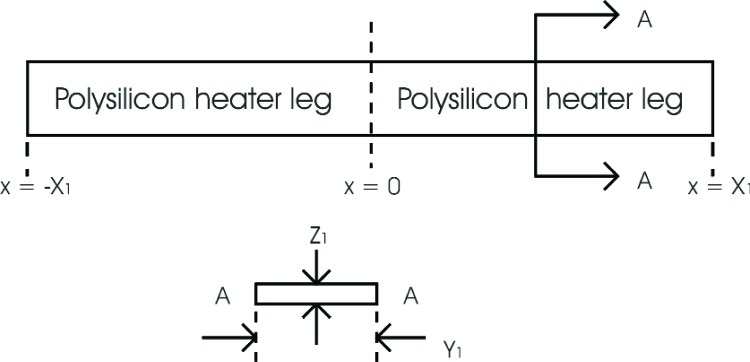
Top and cross-sectional views of a polysilicon heater used as the basis for a microhotplate model with definitions of pertinent design variables.

Consider a homogeneous, rectangular, polysilicon heater leg (heater lead) defined for *X*_1_ ≥ *x* ≥ 0, *Y*_1_ ≥ *y* ≥ 0, *Z*_1_ ≥ *z* ≥ 0 and a second homogeneous rectangular polysilicon heater leg defined for 0 ≥ *x* ≥ −*X*_1_, *Y*_1_ ≥ *y* ≥ 0, *Z*_1_ ≥ *z* ≥ 0 as shown in Fig. 1. Assume that the temperature gradients in the *y* and *z* directions are negligible, let *T*(*x*) be the temperature at the point *x* in the heater leg relative to a reference temperature *T*_1_, and let *ρ*_s_ and *k*_1_ be the resistance per square of the polysilicon, and the thermal conductivity of the polysilicon, respectively.

Further assume that

- heat sinks at temperature *T*_1_ are located at the points *x* = −*X*_1_ and *x* = *X*_1_,
- the leg heaters are connected in electrical series at the plane *x* = 0 through a heater of resistance 2*R*_p_ that simulates the microhotplate platform^1^ heater, and that will be assumed to be isothermal in this paper,
- the thermal conductivity of the polysilicon heater *k*_1_ is replaced by an effective thermal conductivity *k*_e_ that includes the contributions of all of the other components of the heaters legs that are thermally in parallel with the polysilicon heater.

This structure provides a simple model of the thermal performance of a microhotplate with a platform heater. With the simplifications of this model, the resistance of each heater leg is given by

$$R=\rho_{s}\frac{X_{1}}{Y_{1}},$$ (1)

the total resistance of the microhotplate heater is given by

$$R_{h}=2R+2R_{p},$$ (2)

and the effective thermal conductivity is given by

$$k_{e}=\frac{\sum k_{i}Y_{i}Z_{i}}{Y_{1}Z_{1}},$$ (3)

where *k_i_* is the thermal conductivity of the *i*th component in the microhotplate leg, with the subscript 1 already assigned to the polysilicon heater and the subscript 0 assigned to the dielectric layers that encapsulate the microhotplate.

With these assumptions, the power dissipated in a rectangular differential volume d*xY*_1_*Z*_1_ centered on the point *x* in one of the heater legs is given by

$$P=I^{2}\rho_{s}\frac{dx}{Y_{1}}.$$ (4)

The equation describing the steady-state heat flow at the point *x* is

$$\vec{\nabla }\cdot \left(k_{e}\vec{\nabla }T\left(x\right)\right)=k_{e}\frac{d^{2}}{dx^{2}}T\left(x\right)=-\frac{P}{dxY_{1}Z_{1}}=-\frac{I^{2}\rho_{s}}{Y_{1}^{2}Z_{1}},$$ (5)

where *ρ*_s_ and *k*_e_ have been assumed to be independent of temperature [1]. The boundary conditions are given by

$$T\left(-X_{1}\right)=T\left(X_{1}\right)=T_{1}$$ (6)

and

$$\frac{d}{dx}T\left(x\right){|}_{x=0}=\pm \frac{I^{2}R_{p}}{k_{e}Y_{1}Z_{1}},$$ (7)

where the plus sign on the right hand side of Eq. (7) applies for *x* < 0 and the minus sign for *x* > 0. Note that the two boundary conditions represented by Eq. (7) state that half of the power dissipated in the microhotplate platform heater (of resistance 2*R*_p_) flows down each heater leg, as required by the symmetry of the model.

It is readily verified that the solution to Eqs. (5–7) for *x* < 0 is given by

$$T\left(x\right)=T_{1}+\frac{I^{2}X_{1}}{k_{e}Y_{1}Z_{1}}\left(R_{p}\left(1+\frac{x}{X_{1}}\right)+\frac{R}{2}\left(1-\frac{x^{2}}{X_{1}^{2}}\right)\right)$$ (8)

and that the maximum microhotplate temperature for a given heater current *I* occurs at *x* = 0 and is given by

$$T\left(0\right)=T_{1}+\frac{I^{2}X_{1}}{k_{e}Y_{1}Z_{1}}\left(R_{p}+\frac{R}{2}\right).$$ (9)

Equations (8 and 9) show that if any power is dissipated in the heater legs, which will always be the case because *R* cannot be zero, it will contribute to heating the microhotplate platform, but with only half the efficiency of the power dissipated in the platform heater. It is noteworthy in this connection that the conventional trampoline geometry for microhotplates with low-resistance leg heaters in series with a higher-resistance meander heater on a large platform maximizes platform heating relative to leg heating. It is also noteworthy as a consistency check that Eq. (5) above can be transformed into the time independent version of Eq. (1) of Sec. 4.10 (*the equation of conduction of a thin wire heated by an electric current*) in [5], but the further development is quite different because very different applications are being considered.

## 3. Correction for Temperature-Dependence of Heater Resistance

Typically, ab initio thermal models of microhotplates are not very accurate even when the models are implemented in sophisticated finite element solvers. The cause of this problem is that the pertinent thermal properties and especially their temperature dependences are poorly known, vary from one fabrication process to another for nominally identical material, and even vary from device to device when manufactured with the nominally same fabrication process. However, it is possible to make a fairly accurate correction for at least one significant source of error in Eq. (9). Specifically, the electrical resistance at the point *x* in the leg heater is well approximated by

R(T(x))=R[1+αT(x)], (10)

where *α* is the temperature coefficient of electrical resistance of the polysilicon heater, which is approximately constant over a large temperature range. Therefore, the accuracy of Eq. (9) can be improved by the simple step of replacing the platform temperature *R_p_* in Eq. (9) by

$$R_{p}\left(T\left(x\right)\right)=R_{p}\left[1+\alpha T\left(x\right)\right].$$ (11)

Similarly, a correction can be made for the effective temperature coefficient *βα* of the entire polysilicon leg heater, which is defined by

$$R\left[1+\beta \alpha T\left(0\right)\right]=\frac{R}{X_{1}}\int_{-X_{1}}^{0}\left[1+\alpha T\left(x\right)\right]dx$$ (12)

Within the accuracy limitations imposed by other sources of error, substitution into Eq. (12) of the approximate expression for *T*(*x*) given in Eq. (8) yields an excellent approximation for *β*, namely

$$\beta =\left\{\frac{3R_{p}+2R}{6R_{p}+3R}\right\}=\left\{\frac{3\sigma +2}{6\sigma +3}\right\},$$ (13)

where

$$\sigma =\frac{R_{p}}{R}.$$ (14)

Therefore

$$T\left(0\right)\approx \frac{I^{2}X_{1}}{k_{e}Y_{1}Z_{1}}\left\{R_{p}\left[1+\alpha T\left(0\right)\right]+\frac{R}{2}\left[1+\beta \alpha T\left(0\right)\right]\right\}+T_{1}$$ (15)

provides a more accurate approximation than Eq. (9). Note that *σ* is always finite for a micohotplate because there must always be some resistance associated with the leg heaters.

## 4. Solution of Eq. (15) for Current, Voltage, and Power

In this section the microhotplate current, voltage, and power are expressed as a function of the microhotplate-platform temperature in forms useful for designing microhotplates optimized for low-voltage operation. To start, it is convenient to define Δ*T* as the microhotplate temperature in air, which is approximated by

$$\Delta T=C_{a}\left(T\left(0\right)-T_{1}\right)$$ (16)

where *C*_a_ is an empirical constant that accounts for the radiation, air-conduction and air-convection heat losses from the microhotplate because these are not accounted for in Eq. (15). Comparison of measurements of previous microhotplates in vacuum and air have shown that *C*_a_ is approximately constant over a large temperature range, which justifies the use of Eq. (16). The use of Δ*T* emphasizes the fact that *T*(0) is measured relative to *T*(*X*_1_).

Equations (15) and (16) can be combined to give

$$I=\left(\frac{Y_{1}}{X_{1}}\right)\sqrt{\frac{2k_{e}Z_{1}\Delta T}{\rho_{s}\left\{2\sigma \left[C_{a}+\alpha \Delta T\right]+\left[C_{a}+\beta \alpha \Delta T\right]\right\}}}.$$ (17)

Next, Ohm’s law can be used to replace *I* in Eq. (17) by the voltage *V*/*R*_h_, where *R*_h_ is the total heater resistance defined in Eq. (2). The result is

$$V=\sqrt{\frac{8k_{e}\rho_{s}Z_{1}\Delta T}{C_{a}^{2}}\left\{\frac{{\left(\sigma \left[C_{a}+\alpha \Delta T\right]+\left[C_{a}+\beta \alpha \Delta T\right]\right)}^{2}}{2\sigma \left[C_{a}+\alpha \Delta T\right]+\left[C_{a}+\beta \alpha \Delta T\right]}\right\}},$$ (18)

and the product of Eqs. (17) and (18) gives the power dissipated in the microhotplate platform and legs as

$$P=\left(\frac{4k_{e}Z_{1}Y_{1}\Delta T}{C_{a}X_{1}}\right)\left(\frac{\sigma \left[C_{a}+\alpha \Delta T\right]+\left[C_{a}+\beta \alpha \Delta T\right]}{2\sigma \left[C_{a}+\alpha \Delta T\right]+\left[C_{a}+\beta \alpha \Delta T\right]}\right).$$ (19)

Notice that both the microhotplate current *I* and power *P* are inversely proportional to the length *X*_1_ of the microhotplate leg, but that the microhotplate voltage *V* is independent of *X*_1_. Therefore, it is easy to reduce the current or power required to heat a microhotplate to a given temperature by increasing *X*_1_, but the required voltage will remain unchanged. This fact suggests that a microhotplate should first be designed to meet the maximum temperature and voltage requirement imposed by the application and fabrication technology without regard to current and power constraints, and then separately optimized to meet these constraints.

Toward this end, it is very convenient to rewrite Eq. (18) as

$$V=\sqrt{\frac{1}{\varepsilon_{T}\varepsilon_{V}}}V_{0}$$ (20)

where

$$V_{0}=\sqrt{\frac{8k_{1}\rho_{s}Z_{1}\Delta T}{C_{a}}\left\{1+\frac{2\alpha \Delta T}{3C_{a}}\right\}},$$ (21)

$$\varepsilon_{T}=\frac{k_{1}Y_{1}Z_{1}}{\sum k_{i}Y_{i}Z_{i}},$$ (22)

$$\varepsilon_{V}=\varepsilon_{V}\left(\sigma ,\alpha \Delta T/C_{a}\right)=\frac{f\left(0\right)\left[2\sigma +f\left(\sigma \right)\right]}{{\left[\sigma +f\left(\sigma \right)\right]}^{2}},$$ (23)

and

$$f\left(\sigma \right)=\frac{1+\beta \alpha \Delta T/C_{a}}{1+\alpha \Delta T/C_{a}}.$$ (24)

The parameter *V*_0_ in Eq. (20) is the characteristic voltage as a function of platform temperature that is available from a given IC fabrication process. This voltage contains all of the parameters that cannot be varied by the microhotplate designer once the fabrication process has been chosen. The product of *ε*_T_ and *ε*_V_ in the first factor on the right-hand side of Eq. (20) is a figure of merit that contains all of the parameters that can be varied by the microhotplate designer. The parameter *ε*_T_ is the thermal-efficiency of the cross-section design of the microhotplate leg. A microhotplate leg that consists only of a polysilicon heater has the maximum thermal-efficiency, and any other components that are added to the microhotplate leg, such as dielectric encapsulation and temperature-sensor leads, decrease *ε*_T_ from its maximum value of unity by adding parallel conduction paths.

**Fig. 2:**
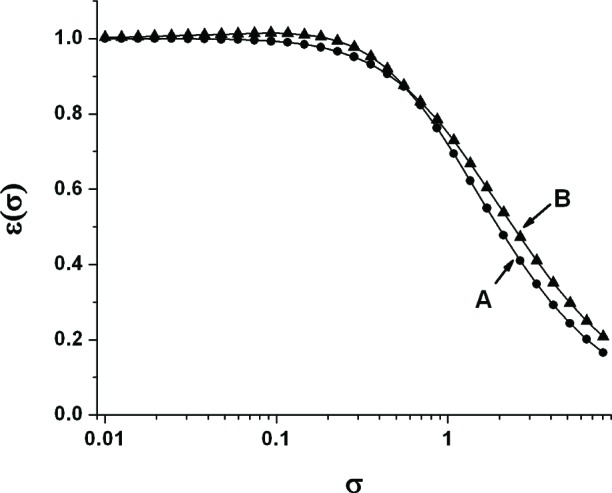
Plot of *ε* versus *σ* for A: *α* Δ*T*/*C*_a_ = 0.01, and for B: *α* Δ*T*/*C*_a_ = 3.1623.

The parameter *ε*_V_ is a figure of merit that describes the efficiency of voltage utilization of the partition of the heater resistance between the microhotplate legs and platform. The range of variation of *ε*_V_ with *σ* is plotted in Fig. 2 for 0.01 < *σ* < 10 with *α* Δ*T*/*C_a_* = 0.01 and *α* Δ*T*/*C*_a_ = 3.1623. This figure illustrates two points about *ε*_V_. First, to obtain the highest temperature from a given voltage, it is important to reduce *σ* below about 0.25, but below this value, the exact value of *σ* makes little practical difference. Second, *ε*_V_ varies little with α Δ*T*/*C*_a_ in the region where *ε*_V_ is near its maximum value. This is the reason why in the design of low-voltage microhotplates, which implies small *σ*, it is practical to treat *ε*_V_ as if it were a parameter set by the choice of fabrication process independent of application (temperature).

The expressions for the microhotplate current and power can also be partitioned into factors that do and do not depend upon the parameters available to the microhotplate designer. Specifically,

$$\begin{array}{l} I=\left(\frac{Y_{1}}{X_{1}}\right)\sqrt{\frac{2k_{e}Z_{1}\Delta T}{\rho_{s}\left(2\sigma \left[C_{a}+\alpha \Delta T\right]+Ca+\beta \alpha \Delta T\right)}}\\ \;=\left(\frac{Y_{1}}{X_{1}}\right)\sqrt{\frac{1}{\varepsilon_{T}\varepsilon_{I}}}I_{0}, \end{array}$$ (25)

and

$$\begin{array}{l} P=\left(\frac{Y_{1}}{X_{1}}\right)\left(\frac{2\left(\sigma +f\left(\sigma \right)\right)}{\varepsilon_{T}\left[2\sigma +f\left(\sigma \right)\right]}\right)\left(\frac{2k_{e}Z_{1}\Delta T}{C_{a}}\right)\\ \;=\left(\frac{Y_{1}}{X_{1}}\right)\left(\frac{1}{\varepsilon_{T}\varepsilon_{P}}\right)P_{0}, \end{array}$$ (26)

where

$$I_{0}=\sqrt{\frac{2k_{1}Z_{1}\Delta T}{\rho_{s}\left[C_{a}+\alpha \Delta T\right]}},$$ (27)

$$P_{0}=\frac{2k_{1}Z_{1}\Delta T}{C_{a}},$$ (28)

$$\varepsilon_{I}=2\sigma +f\left(\sigma \right),$$ (29)

$$\varepsilon_{P}=\frac{2\sigma +f\left(\sigma \right)}{2\left(\sigma +f\left(\sigma \right)\right)},$$ (30)

and *ε*_P_ is the thermal efficiency of the microhotplate design with respect to the partition of heating between the microhotplate legs and platform, and *ε*_I_ is a figure of merit with no obvious simple interpretation. Note that when *σ* << 1, which means that most of the heating takes place in the microhotplate legs, *ε*_P_ ≈ 0.5, but when *σ* >> 1, which means that most of the heating takes place in the platform, *ε*_P_ ≈ 1, which is the factor of two difference in thermal efficiency between platform-only and leg-only heating. Also, note that only the rightmost factor on the right-hand side of Eqs. (20), (25), and (26) is fixed by the IC fabrication process being used and cannot be varied by the microhotplate designer. The other five factors are determined by the microhotplate layout: *X*_1_ and *Y*_1_ are layout variables, *ε*_V_ and *ε*_P_ depend upon the partition of heating between the microhotplate legs and platform, and *ε*_T_ depends upon the microhotplate-leg cross section.

## 5. Design Strategy Based on the Microhotplate Model

Equations (20), (25), and (26) facilitate the design of a microhotplate for low-voltage operation with the following steps:

1. Let Δ*T*_max_, *V*_max_, *P*_max_, and *I*_max_ be the specified maximum values of the temperature difference between the microhotplate platform and the heat sink, the microhotplate voltage, power, and current, respectively.
2. Set *V* and Δ*T* in Eq. (20) to *V*_max_ and Δ*T*_max_, respectively, and solve for *ε*_T_ under the assumption that *σ* = 0, which implies that *ε_V_* = 1.
  - If *ε*_T_ > 1 or is unrealistically close to 1, try a different fabrication process or choose a lower value for Δ*T*_max_.
3. Constrain the microhotplate-leg cross section by a design function *Y*_0_ = *f* (*Y*_1_, *Y*2, …) as illustrated later in this paper.
4. Simultaneously solve Eq. (22) and *Y*_0_ = *f* (*Y*_1_, *Y*2, …) for *Y*_1_ to complete the design of the microhotplate cross section.
5. Set *I* and Δ*T* in Eq. (25) to *I*_max_ and Δ*T*_max_, respectively, and solve for *X*_1I_.
6. Set *P* and Δ*T* in Eq. (26) to *P*_max_ and Δ*T*_max_, respectively, and solve for *X*_1P_.
7. Set *X*_1_ to the maximum of *X*_1I_ and *X*_1P_.
8. Now that an approximate value of *X*_1_ is available, use other design constraints to choose an approximate value of *σ* and repeat steps 2 through 7 to fine tune the design by further changes to *ε*_T_, *σ*, *X*_1_, or *Y*_1_.

As long as *σ* < 0.25, there will be little difference between the results of the first and second iteration, and further iterations will usually be unnecessary. In fact, according to Fig. 2, the voltage performance will usually be somewhat improved, but at the cost of current or power performance. The design process described above is illustrated in the following section, which also compares the measured and predicted performance of a microhotplate designed as described above.

## 6. Example and Model Validation

Consider designing a bridge-type microhotplate in the 0.6 µm MOSIS-AMI C5N [6] CMOS-fabrication process. Assume that the layer properties for this process are those given in Table 1. These value are based on adjusting literature values for the thermal properties of the process layers to give a good fit to results for higher-voltage microhotplates fabricated in the MOSIS-AMI ABN [6] 1.6 µm fabrication process. Further assume that *C*_a_ ≈ 0.7 based on previous measurements of the same microhotplates.

For simplicity, the glass encapsulation is treated as a single layer in Table 1. If sufficient data are available about the individual layers making up the encapsulation, then they can be considered separately, but this will not improve the accuracy of the model predictions without detailed information about the thermal properties and thickness of each layer as a function of the presence or absence of adjacent layers. The Minimum Width *w*_1_ and Minimum Separation *s*_1_ for the Polysilicon in Table 1 are design rules specified by the MOSIS-AMI C5N process. Currently there is no design rule for the Minimum Separation *s*_0_ for the Glass Encapsulation, which is the minimum distance between a polysilicon structure and an open area [4] that is used to expose bare silicon for releasing CMOS MEMS structures, but design rules for this distance will be needed. The choice of *s*_0_ = 7.8 µm is based on past experience, but is probably rather conservative.

Next, assume that the design goal is *T*_max_ = 500 °C above ambient with *V*_max_ < 3 V, *I*_max_ < 10 mA, and *P*_max_ < 20 mW. With this information as well as the information in Table 1, it is possible to calculate *V*_0_, *I*_0_, *P*_0_, and the minimum required value for the product *ε*_T_*ε*_V_. The results are given in Table 2 along with other intermediate values. In Table 2, *d* = 2*w*_1_ = 2*s*_1_ is twice the minimum design-rule width and separation between polysilicon lines for the chosen fabrication process, and *D* = *s*_0_ is the minimum distance between a polysilicon line and the open area. The parameter *d* could have been set to the minimum width and separation between polysilicon lines for the chosen fabrication process for this example, but maximum current density limitations at the maximum platform temperature *T*_max_, which do not currently exist, will have to be satisfied in practice. These may even have to be temperature dependent. Also, as mentioned above, there are currently no design rules for *D* in standard CMOS-IC fabrication processes, but they will also be needed for each submicron process that is qualified for embedded-sensor SoC fabrication, and they may also have to be temperature dependent.

**Fig. 3:**
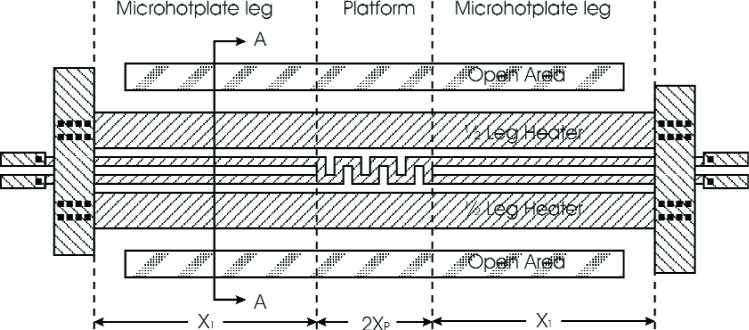
Top view of a bridge-type microhotplate that is modeled here including the assignment of the model design variables.

A top view of a proposed design for the bridge-type microhotplate is shown in Fig. 3. Notice that the microhotplate heater has a platform and two identical legs having uniform cross section along the long axis of the legs. There is no precise definition for the location of the boundary between the microhotplate platform and legs at the level of approximation employed in this model. The basic idea is that the platform should be approximately isothermal and that any deviations from isothermality are not described by the model. The symmetry of the microhotplate minimizes the thermal gradients around *x* = 0, so the platform should be centered around this point. Any additional platform components such as temperature sensors, sensing films (not shown), and electrodes (not shown) for making electrical connection to sensing films will contribute parallel thermal conduction paths that will reduce the thermal gradients in the platform.

**Fig. 4:**
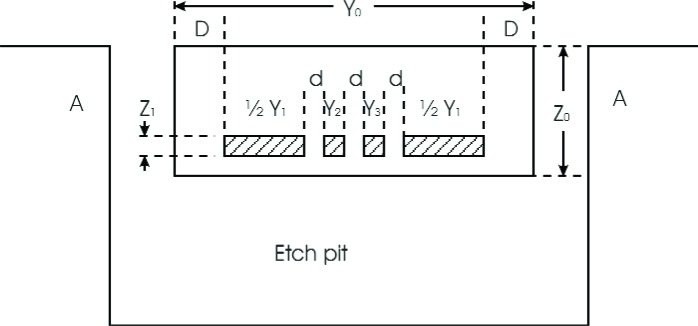
Cross-sectional view of a bridge-type microhotplate that is modeled here including the assignment of variables to the model developed here.

The portions of the heater located in the microhotplate legs are the leg heaters, and the portions located in the platform are the platform heaters. Notice that the heater consists of two electrically parallel strips of polysilicon, each of which constitutes half of the heater. Figure 4, which shows a cross sectional view of one of the microhotplate legs shown in Fig. 3, presents a convenient partition of the microhotplate leg into components based on the data given in Table 1.

According to Fig. 4, *Y*_0_ is constrained to satisfy the design function

$$Y_{0}=f\left(Y_{1},Y_{2},Y_{3}\right)=2D+3d+Y_{1}+Y_{2}+Y_{3}.$$ (31)

To continue the design, let

$$Y_{2}=Y_{3}=d,$$ (32)

as discussed above. Therefore,

$$\begin{array}{l} \varepsilon_{T}=\frac{k_{1}Y_{1}Z_{1}}{k_{0}Y_{0}Z_{0}+\left(k_{1}-k_{0}\right)\left(Y_{1}+Y_{2}+Y_{3}\right)Z_{1}}\\ \approx \frac{k_{1}Y_{1}Z_{1}}{k_{0}Y_{0}Z_{0}+k_{1}\left(Y_{1}+Y_{2}+Y_{3}\right)Z_{1}}, \end{array}$$ (33)

where the approximation on the right-hand side of Eq. (33) provides a design margin.

Tables 3 and 4 present the results of two iterations of the design process following the steps outlined above. Based on the results of the first iteration, it was decided to use *w*_1_ and *s*_1_ to define the dimensions of the polysilicon meander in the platform temperature sensor while maintaining the geometry shown in Fig. 3, and to set *X*_1_ = 85.5 µm. These choices set *X*_P_ = 5.4 µm, which made *σ* = 0.05614.

Once preliminary values for *Y*_1_, *Y*_0_, *X*_1_, and *σ* were available, the second design iteration, the results of which are listed in Table 4, was carried out. These tables illustrate a number of important points. First, the model developed in this paper predicts that it will be possible to obtain useful temperatures with CMOS microhotplates designed for low-voltage operation. Second, the length of the heater leg decreased a little during the second iteration, but not enough to make a third iteration with a new value of *σ* and *X*_1_ seem worthwhile. Had it increased, which can occur with larger values of *σ*, more iterations may be required. Third, the values chosen for *X*_1_, *Y*_1_, *Y*_0_ after the first iteration are all integer multiples of *w*_1_/2 = 0.3 µm as required when laying out the microhotplate design for the MOSIS-AMI C5N process.

The final microhotplate dimensions were chosen to be a little more conservative than those given in Tables 3 and 4, and a microhotplate with the dimensions given in the caption to Table 5 was fabricated and tested. The first test was to calibrate the temperature sensor as described in Ref. [2]. The second test was to force different currents through the microhotplate heater and to measure the resulting heater voltage and platform temperature, the latter being calculated from the temperature-sensor calibration. The results are given in Table 5. The ratios of the measured temperatures and voltages to the predicted temperatures and voltages are also given in the table. The ratios of the measured to predicted powers are not included in the table because they are identical to the voltage ratios to within rounding error because the theoretical and experimental input currents are identical.

The agreement between the predicted and measured results are not particularly good, but they are good enough to provide a practical design for the microhotplate. Specifically, the design goals, namely that *T*_max_ = 500 °C above ambient with *V*_max_ < 3 V, *I*_max_ < 10 mA, and *P*_max_ < 20 W, were all met. On the other hand, it may be desirable to have a model that can predict microhotplate thermal performance much more accurately than the results given in Table 5.

It is not known whether the differences between the predicted and measured results shown in Table 5 are caused by errors in the thermal properties given in Table 1 or by the simplifying assumptions that were used to derive the model, or both. In the first case, it would be possible to obtain improved values of the thermal properties with a least-squares fit of the model predictions to experimental data measured on a number of microhotplates fabricated in the same process but having different values of *X*_1_, *Y*_1_, and *Y*_0_. Furthermore, once optimum values for the thermal properties are available, the model could be used for simulating microhotplate performance in SoC design projects. If, on the other hand, the differences in Table 5 are caused predominately by defects in the model, it should be possible to improve the model by adding empirical correction factors whose parameters could also be optimized by a least-squares fit to experimental data. In any case, a model will be needed for SoC design simulations, and least-squares fitting data from at least a small number of designs that span a practical design space will be necessary to improve and/or to validate the model, as well as to characterize its uncertainty.

**Fig. 5:**
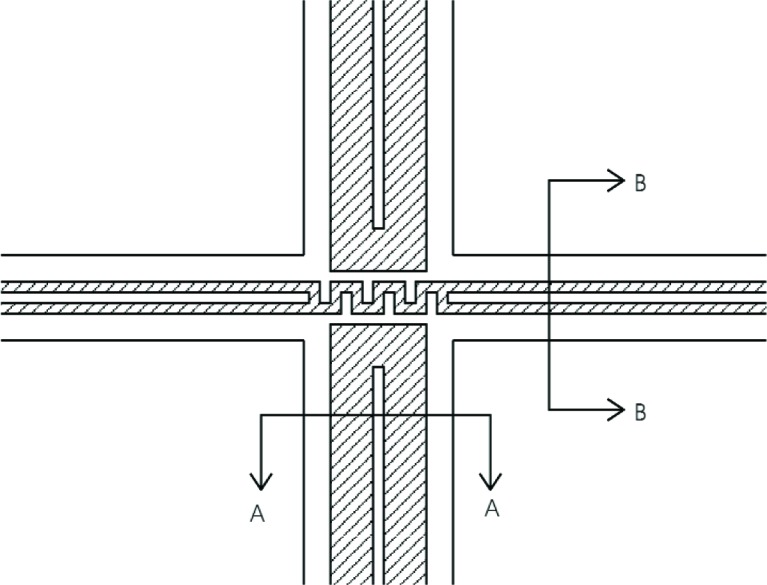
Top view of a trampoline-type microhotplate and the assignment of variables for possible future application of the model developed here.

**Fig. 6:**
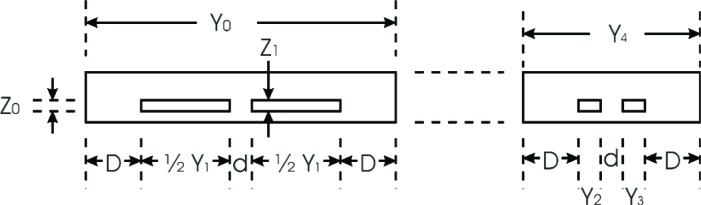
Cross-sectional view of a bridge-type microhotplate and the assignment of variables for possible future application of the model developed here.

The model derived here can also be applied to trampoline-type microhotplates having two sets of identical legs as illustrated in Figs. 5 and 6. Figure 6 shows appropriate, but non-unique, assignments to the variables *Y*_0_, *Y*_1_, etc. Note that the structure consisting of two non-identical legs is treated as a single leg in the model so that the width of the leg in the model is given by *Y*_0_ + *Y*_4_. It is beyond the scope of this paper to carry out a comprehensive comparison of bridge-type and trampoline-type microhotplates, but two general conclusions are apparent. First, if bridge-type and trampoline-type microhotplates are designed to be as similar as possible, then the bridge-type will occupy less area due to its intrinsically more compact geometry. Secondly, if the design rules are such that *D* ≫ *d*, as will probably be the case, then *ε*_T_ for the bridge-type will be somewhat smaller than *ε*_T_ for the trampoline-type due to the presence of the two extra microhotplate legs in the trampoline-type. For instance, compare Eq. (31) with

$$Y_{0}+Y_{4}=4D+2d+Y_{1}+Y_{2}+Y_{3},$$ (34)

which increases the denominator in the equation defining *ε*_T_ [Eq. (22)] by *k*_0_(2*D* − *d*) provided that *D* > *d*/2.

## 7. Summary and Conclusion

A simple, approximate model of microhotplate thermal performance has been derived to assist the design of microhotplates to meet the requirements of low-voltage applications. The model shows that confining heating to the microhotplate platform is the most efficient use of the available electrical power, but confining the heating to the microhotplate legs is the most efficient use of the available voltage. The model also shows that the voltage required to obtain a given microhotplate-platform temperature is approximately independent of the length of the microhotplate heater legs, particularly when the platform-heater resistance is small compared to the leg-heater resistance. On the other hand, both the microhotplate current and power are approximately inversely proportional to the length of the microhotplate heater legs. The model further shows that bridge-type microhotplates are more thermally efficient than trampoline types, all other parameters being equal. Finally, in low-voltage applications it is possible to first design the cross section of the microhotplate legs to meet the temperature versus voltage constraints on the microhotplate and then to choose the leg length to meet the temperature versus current and power constraints simultaneously. But a few iterations may be required to meet all of the constraints.

A number of metrology and standards issues became apparent during the development of the model described here. First, standard test structures from which the pertinent thermal properties for microhotplate design can be extracted will be necessary if more accurate predictions of all microhotplate properties are desired, particularly when other structures like sensor-film electrodes are to be incorporated in the microhotplate legs. Second, new design rules for the spacing between structures made from different standard CMOS layers such as polysilicon-polysilicon and polysilicon-open areas as a function of the maximum microhotplate platform temperature will be needed to support the development of microhotplate-based virtual components for SoC design. Finally, standards for the long-term stability of the electro-thermal properties of the different standard CMOS layers, which are also likely to depend upon the platform temperature versus time history, will also be needed.

## Figures and Tables

**Table 1:** Nominal microhotplate parameters for the MOSIS-AMI C5N 0.6 µm CMOS process

Layer	Polysilicon (*i* = 1)	Glass encapsulation (*i* = 0)
Thickness, *Z_i_*	0.4 µm	3.2 µm
Thermal Conductivity, *k_i_*	0.65 W/cm·K	0.014 W/cm·K
Resistance per square, *ρ*_s_	25 Ω/square	∞
Temperature Coefficient of Resistance, *α*	0.0011	Not applicable
Minimum Width, *w_i_*	0.3 µm	Not applicable
Minimum Separation, *s_i_*	0.3 µm	7.8 µm

**Table 2:** Design parameters fixed by the specific application and fabrication technology given in Table 1

Parameter	Value	Unit
*T*_m_	500	K
*V*_m_	3.0	V
*I*_m_	10.0	mA
*P*_m_	20.0	mW
*V*_0_	2.38	V
*I*_0_	0.0289	mA
*P*_0_	0.0743	mW
*ε*_T_*ε*_V_	0.627	
*d*	1.2	µm
*D*	7.8	µm

**Table 3:** Example of the first iteration of the design process described in this paper based on the assumption that *σ* = 0

Parameter	Value	Unit
*Σ*	0.0	
*ε*_V_	1.000	
*ε*_T_	0.629	
*Y*_1_	14.65	μm
*Y*_0_	36.25	μm
*X*_1I_	86.5	μm
*X*_1P_	57.7	μm
*X*_1_	86.5	μm
Δ*T*	500	°C
*V* (*T*)	3.00	V
*I* (*T*)	6.67	mV
*P*(*T*)	20.00	mW

**Table 4:** Example of the second iteration of the design process described in this paper based on the assumption that *σ* = 0.0514

Parameter	Value	Unit
*Σ*	0.05614	
*ε*_V_	1.005	
*ε*_T_	0.626	
*Y*_1_	14.4	μm
*Y*_0_	36.0	μm
*X*_1I_	53.6	μm
*X*_1P_	80.4	μm
*X*_1_	80.4	μm
Δ*T*	500	°C
*V* (*T*)	3.00	V
*I* (*T*)	6.67	mV
*P*(*T*)	20.00	mW

**Table 5:** Comparison of the temperature difference, voltage, and power as a function of applied current as predicted (subscript *p*) with the model described here and as measured (subscript m) for a microhotplate having the properties given in Table 1 with *σ* = 0.06316, *X*_1_ = 85.5 μm, *Y*_1_ = 15.0 μm, *Y*_0_ = 36.0 μm, *d* = 1.2 μm, and *D* = 7.8 μm

*I* (mA)	Δ*T*_p_ (K)	Δ*T*_m_	Ratio	*V*_p_(V)	*V*_m_(V)	Ratio	*P*_p_(mW)	*P*_m_(mW)
1.000	8	6	0.75	0.306	0.292	0.954	0.306	0.292 (mW)
1.500	18	15	0.83	0.463	0.439	0.948	0.695	0.658 (mW)
2.000	32	28	0.88	0.627	0.590	0.940	1.253	1.181 (mW)
2.500	52	45	0.87	0.799	0.747	0.935	1.997	1.868 (mW)
3.000	76	67	0.88	0.982	0.910	0.927	2.946	2.730 (mW)
3.500	107	95	0.89	1.180	1.081	0.916	4.129	3.784 (mW)
4.000	145	128	0.88	1.396	1.264	0.905	5.586	5.054 (mW)
4.500	191	170	0.89	1.638	1.459	0.891	7.369	6.564 (mW)
5.000	248	220	0.89	1.910	1.671	0.875	9.550	8.354 (mW)
5.500	318	283	0.89	2.224	1.905	0.857	12.23	10.48 (mW)
6.000	405	359	0.89	2.592	2.165	0.835	15.55	12.99 (mW)
6.500	514	455	0.89	3.034	2.462	0.811	19.72	16.00 (mW)
7.000	655	564	0.86	3.581	2.803	0.783	25.07	19.61 (mW)

## Footnotes

1 Elsewhere, “platform” is also used to distinguish the microhotplate structure from a more complex structure that includes the microhotplate structure as a component, such as a microhotplate-based gas-sensor.
